# Liquid-Based Medium Used to Prepare Cytological Breast Nipple Fluid Improves the Quality of Cellular Samples Automatic Collection

**DOI:** 10.14740/wjon844e

**Published:** 2014-08-25

**Authors:** Marco Antonio Zonta, Fernanda Velame, Samara Gema, Jose Roberto Filassi, Adhemar Longatto-Filho

**Affiliations:** a Infectology Department, Faculty of Medicine, Federal University of Sao Paulo; Santo Amaro University, Brazil; b IN CITO - Citologia Diagnostica Lab, Sao Paulo, Brazil; c Mastology Section, Department of Gynecology, Faculty of Medicine, Sao Paulo University, Brazil; d Laboratory of Medical Investigation (LIM) 14, Department of Pathology, Faculty of Medicine, Sao Paulo University, Brazil; e Life and Health Sciences Research Institute (ICVS), School of Health Sciences, University of Minho, Braga, Portugal; ICVS/3B’s - PT Government Associate Laboratory, Braga/Guimaraes, Portugal; Molecular Oncology Research Center, Barretos Cancer Hospital, Barretos, Sao Paulo, Brazil; f Molecular Oncology Research Center, Barretos Cancer Hospital, Pio XII Foundation, Barretos, Brazil

**Keywords:** Breast cytology, Liquid based cytology, Atypical ductal cells, Ductal carcinoma

## Abstract

**Background:**

Breast cancer is the second cause of death in women worldwide. The spontaneous breast nipple discharge may contain cells that can be analyzed for malignancy. Halo^®^ Mamo Cyto Test (HMCT) was recently developed as an automated system indicated to aspirate cells from the breast ducts. The objective of this study was to standardize the methodology of sampling and sample preparation of nipple discharge obtained by the automated method Halo breast test and perform cytological evaluation in samples preserved in liquid medium (SurePath™).

**Methods:**

We analyzed 564 nipple fluid samples, from women between 20 and 85 years old, without history of breast disease and neoplasia, no pregnancy, and without gynecologic medical history, collected by HMCT method and preserved in two different vials with solutions for transport.

**Results:**

From 306 nipple fluid samples from method 1, 199 (65%) were classified as unsatisfactory (class 0), 104 (34%) samples were classified as benign findings (class II), and three (1%) were classified as undetermined to neoplastic cells (class III). From 258 samples analyzed in method 2, 127 (49%) were classified as class 0, 124 (48%) were classified as class II, and seven (2%) were classified as class III.

**Conclusion:**

Our study suggests an improvement in the quality and quantity of cellular samples when the association of the two methodologies is performed, Halo breast test and the method in liquid medium.

## Introduction

The evaluation of methods to assist the clinical prediction of proliferative breast disease has been the objective of health professionals and specifically the ones focused on the early detection and prevention of breast cancer. Breast cancer is the second cause of death in women worldwide and, in Brazil there were approximately 53,000 new cases in 2012 [1].

Screening of breast cancer can be done by clinical examination and mammography, and Brazilian Public Health Authorities indicate this protocol for women who are older than 40 years old; despite the quality inherent to the mammogram test, the mortality rate is still high [2-4].

The spontaneous breast nipple discharge is characterized by the secretion of fluid through the breast nipples coming from mammary ducts. This material may contain cells that can be analyzed for malignancy. The study of this material is valuable for assessing possible cellular alterations associated to the malignant transformation in breast tissue, including precursor lesions [5-11].

Halo^®^ Mamo Cyto Test (HMCT) was recently developed as an automated system indicated to aspirate cells from the breast ducts. The papillary fluid is obtained by a support adapted to the Halo system in order to promote the suction of the fluid material for cytological analyses. When preserved in appropriate liquid medium, as those used for uterine cervix liquid-based cytology (LBC), it is possible to analyze genomic and proteomic contents of the breast sample (data not published). This methodology emerges as a new alternative to obtain cells for the identification of possible atypical in early stages. It is easy to be used and typically tolerable for the patients [12].

The LBC that unquestionably offers better cytological preparations for gynecological samples is thought to be also better for breast material in terms of best cellular preservation. Also, as mentioned previously, the residual material can be used for molecular analyses proposals [13].

The objective of this study was to standardize a methodology of sampling and sample preparation of nipple discharge obtained by the automated method Halo breast test and perform cytological evaluation in samples preserved in liquid medium (SurePath™).

## Methods

We analyzed 564 nipple fluid samples, from women between 20 and 85 years old, without history of breast disease and neoplasia, no pregnancy, and without gynecologic history collected by HMCT method and preserved in two different vials solutions for transport. From the 564 samples, 306 were preserved in 8% solution of ethanol and 258 were preserved in SurePath™ vials for automatic processing. The project has been approved by the Ethics Commission for Analysis of Research Projects (CAPPesq) and by the Ethics Committee of the Department of Obstetrics and Gynecology, Faculty of Medicine, University of Sao Paulo, on May 27, 2008 (Table 1).

**Table 1:** Demographics and Social Status of Women Included in the Study

	Group I (159 women)	Group II (130 women)
Age	20 - 85	20 - 85
Breast feeding		
Yes	112 (70.44%)	112 (86.16%)
No	47 (29.56%)	18 (13.84%)
Family history of breast disease		
Yes	99 (62.27%)	31 (23.84%)
No	60 (37.73%)	99 (76.16%)
Nipple discharge		
Yes	67 (42.14%)	33 (25.39%)
No	92 (57.86%)	97 (74.61%)
History of breast disease		
Yes	5 (3.15%)	31 (23.85%)
No	154 (96.85%)	99 (76.15%)

### Automated nipple fluid papillary

Women were invited to participate in the project and the protocol was explained. After reading, agreeing and signing the term of informed consent, a questionnaire was conducted to obtain clinical and epidemiological data.

The procedures were performed in two stages, the first method was performed at the Gynecology Department of the Faculty of Medicine, University of Sao Paulo, Brazil and the material was preserved in medium based on ethanol and buffered. In the second stage, the material was collected at the Gynecology Department of Military Hospital of the Rio de Janeiro city, Brazil and preserved in SurePath™ medium.

**Figure 1:**
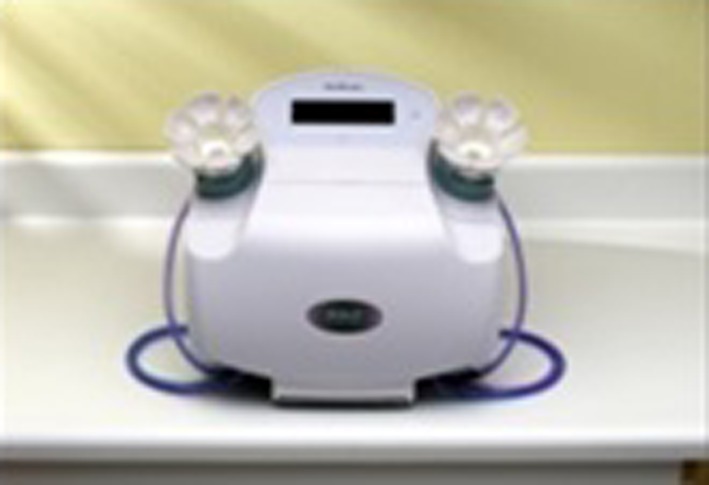
HAL breast test equipment.

**Figure 2:**
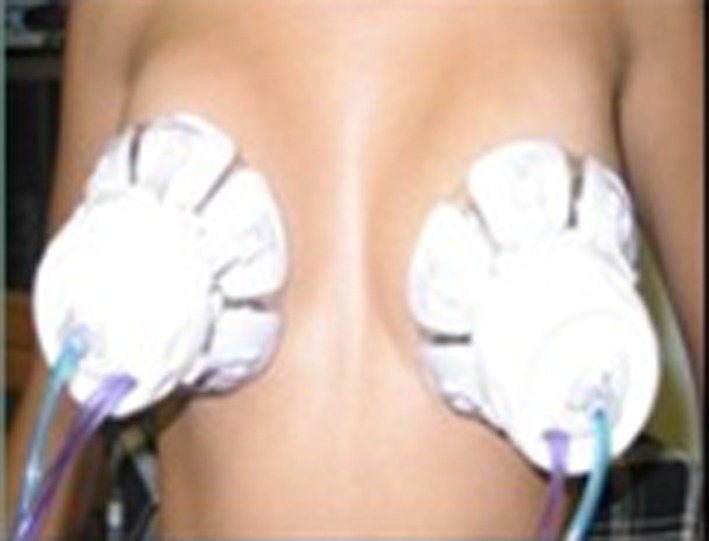
Disable silicone cups, “Tulips”, used for collecting fluid samples papillary.

**Figure 3:**
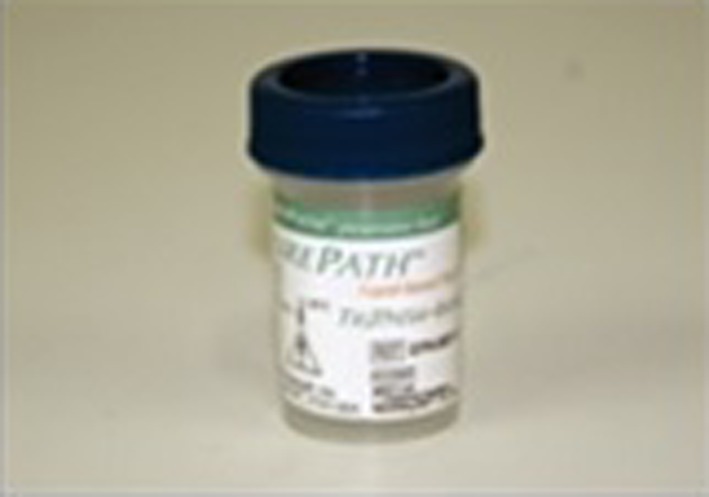
Vial with liquid to preserve the cellular samples (SurePath™).

In both arms, women were positioned to perform the collection procedure. A self-massage was applied for 10 min in each breast, starting the armpits and ending at the nipple. After the self-massage was completed, the cups that were supported around the nipple and aspiration cycle comprised of a light pressure and vacuum at an approximately 40 °C temperature were placed in order to obtain fluid material. The cups are composed of a disposable biocompatible polymer (silicone). In sequence, we had the Halo cups opened and adjusted according to the size of the breasts on the areola region. The suction procedure of 5 min aspiration cycle was performed. After the procedure, we transferred the nipple fluid into vials and SurePath medium. The residual nipple fluid present in the areola region was collected using a swab and maintained into two respective recipients (Fig. 1-3).

### Cytological preparation methodology (method 1)

From 306 nipples fluid sample obtained from right and left breasts and preserved in methanol. All the cellular material (1 mL) was subjected to vortex process for 15 s at 3,000 rpm. The cellular suspension was transferred to a conical tube and centrifuged for 10 min at 800 g to get a “pellet” of cells.

Tris buffer (Sigma) (1 mL) was added in the pellet that was homogenized and 1 mL was transferred to the slides with positive charge used to fix cells for 10 min. After the settling time (10 min) and cell attachment, material was decanted and washed with isopropyl alcohol and submitted to the automated staining.

### Modified cytology method (method 2)

Two of the authors (MZ and FV) processed part of the samples using a new protocol methodology where 306 were firstly preserved in 8% solution of methanol. Two hundred and fifty-eight (258) samples of the right and left breasts from 130 women were collected by automated HMCT. After preserving samples in a SurePath™ vial, the samples were subjected to a suspension process (vortex) for 15 s at 3,000 rpm. Two milliliter of all samples was transferred to conical tube. The samples were centrifuged for 10 min at 800 g for cell pellet formation. The supernatant was removed and completed with 1 mL Tris buffer (Sigma) for homogenization, 0.1 mL solution was transferred to delimit field area with slides charged positively for 20 min in order to have settlement and cell attachment; after that, the material was decanted and washed with isopropyl alcohol and again decanted to automate staining (Table 2).

**Table 2:** Methods Used for Obtaining Cellular Material for Cytological Analysis

	Method 1	Method 2 (modified by Zonta & Velame)
Preserved medium	Methanol 8%	SurePath ethanol-based medium
Technical procedure	1 mL of sample fluid	2 mL of sample fluid
	Vortex 15 s/3,000 rpm	Vortex 15 s/3,000 rpm
	10 min at 800 g	10 min at 800 g
	Cells pellet	Cells pellet
	1 mL of Tris buffer (Sigma)	1 mL of Tris buffer (Sigma)
	1 mL of homogeneous cells pellet	1 mL of homogeneous cells pellet
	10 m slyde fixing	20 m slyde fixing

### Automated staining

The samples were stained in automated staining equipment (Prepmate™, BD, Burlington, USA). The cytological preparations were classified according to the National Statistics and The National Health Service Breast Screening Programme [14] (Table 1).

## Results

**Figure 4:**
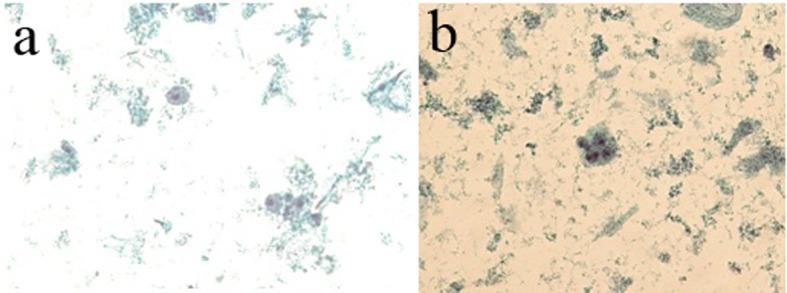
(a) The presence of foam cells, inflammatory cells and cellular debris in basophilic proteinaceous background. (b) The presence of ductal cells with increased nucleus/cytoplasm ratio and hyperchromatism nuclear and acidophilic cytoplasm. Presence of foam cells, mononuclear cells and cellular debris in basophilic proteinaceous background (method 1 - automated Papanicolaou, × 40).

From 306 nipple fluid samples from method 1, 199 (65%) were classified as unsatisfactory (class 0); no cellular representation was observed. These acellular samples exhibited a hyaline proteinaceous background. One hundred seven (35%) samples were classified as benign findings (class II). The smears were characterized by the presence of uniform groups of ductal cells, with round nuclei and single small nucleoli. The nuclear membrane showed regular borders and the cytoplasmic membrane showed well basophilic borders. Inflammatory mononuclear and multinucleated cells, ductal cells and foam cells were observed in proteinaceous background. Three (1%) samples showed cellular features with nuclear atypia, including nuclear hyperchromasia, discrete irregular chromatin, increased nucleus/cytoplasm ratio, and irregular borders of nuclear membrane and were classified as undetermined to neoplastic cells (class III) (Table 3, Fig. 4a, b).

**Table 3:** Cytological Classification for Breast Screening Program

Class 0	Unsatisfactory material: absence of cells
Class II	Negative for malignancy
Class III	Indeterminate for malignancy
Class IV	Suspicious for malignancy
Class V	Positive for malignancy

National Statistics and The National Health Service Breast Screening Programme (NHSBSP)

**Figure 5:**
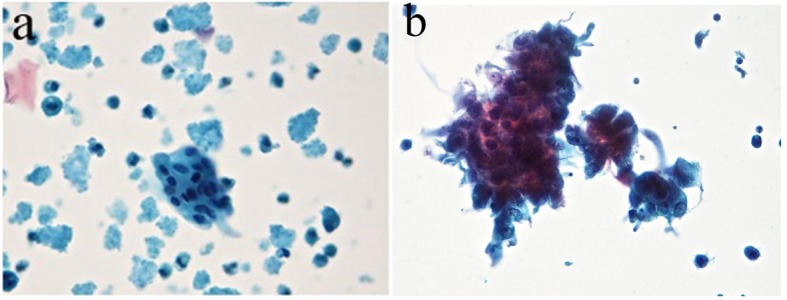
(a) Ductal cells within the normal range in proteinaceous hyaline background. Absence of interfering cell and cellular debris was observed. (b) The presence of atypical ductal cells with hyperchromatic nuclei, dark chromatin with granular deposit, irregular nuclear membrane, single prominent nucleoli, and a group of cellular overlapped. Cellular clusters, foam cells and mononuclear preserved in hyaline proteinaceous background were observed. Absence of interfering and debris cellular were observed (Zonta & Velame method - Papanicolaou automated, × 40).

From 258 samples analyzed in method 2, 127 (49%) had no cellularity and were classified as class 0, and 124 (48%) had cellularity with benign features and classified as class II. Ductal cells with cytomorphological characteristics within normal features were observed; there were also foam cells dispersed in hyaline proteinaceous background. Seven (2%) samples showed ductal cells with atypical nuclei showing hyperchromatic nuclei and irregular nuclear membrane and chromatin condensation were observed. The nucleoli were increased and hyperchromatic. The cytoplasms of the cells were frequently acidophilic and finely vacuolated. Cell clusters with nuclear overlapping (class III) were observed. The smear background showed polymorphonuclear cells, blood cells in proteinaceous background (Table 4, Fig. 5a, b).

**Table 4:** Nipple Fluid Samples Collected in an Alcoholic Medium and Subjected to Manual Preparation and Stained by Automated Papanicolaou Method

Cellular changes	Frequency (%)
Unsatisfactory	199 (65.0%)
Benign cells - class II	104 (34.0%)
Atypical cells - class III	3 (1.0%)
Total	306 (100%)

From 306 nipple fluid samples prepared by method 1, a higher frequency of benign lesions (61/19.93%) in women between 46 and 55 years old was observed. The major number of unsatisfactory (85/27.77%) samples was observed in the same age group. Three (0.96%) nipple fluid samples with atypical ductal cells, one (0.32%) from women between 46 and 55 years old, one (0.32%) in sample from women between 56 and 65 year old and one (0.32%) sample from women between 66 and 75 years old (Table 5) were observed.

**Table 5:** Cell Samples Prepared by the Automated Method (Zonta & Velame) and Stained by Automated Papanicolaou Method

Cellular changes	Frequency (%)
Unsatisfactory - class 0	127 (49.0%)
Benign cells - class II	124 (48.0%)
Atypical cells - class III	7 (3.0%)
Total	258 (100%)

Concerning the nipple fluid sample prepared in method 2, a higher frequency of benign finding (37/13.34%) in women between 46 and 55 years old and women between 36 and 45 (32/12.40%) years old was observed (Table 6).

**Table 6:** Frequency of Benign and Atypical Lesions in Women Evaluated by Method 1 (306 Sample, 100%)

Women age (method 1)	Unsatisfactory (%)	Benign cells (%)	Atypical cells (%)
	Class 0	Class II	Class III
20 - 25	5 (1.63%)	3 (0.98%)	0 (0%)
26 - 35	10 (3.27%)	8 (2.61%)	0 (0%)
36 - 45	49 (16.01%)	12 (3.92%)	0 (0%)
46 - 55	85 (27.78%)	61 (19.93%)	1 (0.33%)
56 - 65	41 (13.40%)	13 (4.25%)	1 (0.33%)
66 - 75	8 (2.61%)	4 (1.31%)	1 (0.33%)
76 - 85	1 (0.33%)	3 (0.98%)	0 (0%)
Total of samples	199 (65.03%)	104 (33.98%)	3 (0.99%)

Also, a higher frequency of atypical cells (class III) (3/1.16%) was observed in women between 36 and 55 years, and two (0.77%) samples in women between 46 and 55 years; one case (0.38%) in a woman between 26 and 35 years old and one (0.38%) between 76 and 85 years old. Unsatisfactory samples were observed in 45 (17.44%) samples from women between 46 and 55 years old (Table 7).

**Table 7:** The Frequency of Benign and Atypical Lesions in Women Evaluated With Method 2

Women age (method 2)	Unsatisfactory (%)	Benign cells (%)	Atypical cells (%)
	Class 0	Class II	Class III
20 - 25	6 (2.32%)	4 (1.55%)	0 (0%)
26 - 35	15 (5.81%)	14 (5.43%)	1 (0.39%)
36 - 45	27 (10.46%)	32 (12.40%)	3 (1.16%)
46 - 55	45 (17.44%)	37 (13.34%)	2 (0.77%)
56 - 65	13 (5.04%)	25 (9.69%)	0 (0%)
66 - 75	12 (4.65%)	6 (2.32%)	0 (0%)
76 - 85	9 (3.49%)	6 (2.32%)	1 (0.39%)
Total of samples	127 (49.17%)	124 (48.05%)	7 (2.71%)
Total		258 (100%)	

## Discussion

The standardization of methodologies which increases the sensitivity and specificity in diagnosis is an important tool for the prevention and diagnosis of breast cancer. A good performance to obtain and preserve cellular material shall increase the possibility of finding cellular changes in initial stages, increasing the sensitivity of the findings in preventions programs.

The use of methods which cause a little or no discomfort when obtaining samples for prevention and diagnoses and provide a better preservation of samples to be analyzed is of great value in early diagnosis of lesions with malignant potential.

The Halo breast test is a methodology that obtains cellular material from the breast ducts aspirating from nipple while causing minimal discomfort to women. This methodology, when compared to mammography and fine-needle aspiration cytology (FNAC) method proves to be significantly less painful, and it has been compared to the act of breastfeeding. The score of pain reported by women is around 3.1. The score of pain reported by women who did the FNAC test and mammography was around 8.0 [15, 16].

The cytological test (Pap test) is a reference for identifying early lesions of the cervix, using the Papanicolaou method. The application of this methodology in other sites can provide important information, promoting the identification of precursor lesions. The use of LBC showed an increase in quality of cellular samples, because preservation of the cells in a liquid solution could be improved. The preparation and stain samples can be automated. This procedure could be used as an alternative for identification of possible changes in cells in early stages, without signal of breast disease, anticipating procedures such as fine-needle aspiration (FNA) and mammography.

Nipple breast fluid can offer a rich cellularity for analyses. The ones present in ductal system normally are exfoliated inside the duct and are eliminated as nipple fluid. The study of cellular material is very important because cellular change in this material can be identified and, cellular evidence of malignity can also be identified. Lesions in initial stage can be obtained and identified in a little nipple secretion, often preceding the clinical diagnosis and mammography analysis [17, 18].

This aspect of the fluid may be an indicative of malignant disease of the breast. The presence of crystalline or bloody material in either unilateral or bilateral nipple discharge, with single ductal conduit affected and persistent secretion are indicators of malignant breast disease. Although a small percentage (2-2.5%), women who have these characteristics are diagnosed with a malignant disease of the breast [19, 20].

Material from nipple discharge showed around 35% of sensitivity to identify precancerous lesion a malignant cells for breast cancer, and 70% of this material is classified as suspicious, because they showed important nuclear changes with atypical forms and size. This could lead to screening FNA providing preliminary results, increasing the accuracy in the diagnosis of breast diseases [21, 22].

The standardization of methodology for studying this material provides a breakthrough not only in the early identification of breast lesions, but also the creation of control programs, that can be applied in populations at risk of developing the disease.

The best preservation of cellular material obtained by FNAC or nipple aspirate increased the diagnostic sensitivity. Mygdakos et al, comparing cell samples of breast, thyroid and salivary glands, found improvements of cytomorphological characterization of the samples prepared in liquid medium compared to the conventional method. It is also important to highlight the necessity of having better training for the professionals who are involved in cytological diagnosis [23].

Our study showed two distinct profiles, the one who had collected material prepared by the conventional method, showed the presence of degenerated ductal cells, with loss of nuclear and cytoplasmic characteristics. A background smear showed a hyaline amorphous material filled with cellular elements degenerated.

There was a high rate of unsatisfactory samples, suggesting that the lack of standardization when preparing smears can be decisive in the loss of quality of the cells. In cases classified as atypical, it was difficult to observe some important nuclear characteristics, such as hyperpigmentation marked irregularity of the nuclear membrane due to virtue of the degree of degeneration of the smears [24].

Samples can be well observed in smears collected by FNAC, which have a large amount of red blood cells and cellular debris due to the technique of harvesting the material. The insufficient fixation and overlapping artifacts on cells with possible morphological changes may hinder the cytological diagnosis [13].

The association between Halo breast test and cellular material was preserved in liquid (SurePath), and it was observed that cell morphology is maintained. The ductal cells had evident nuclear features well distributed in chromatin, and prominent nucleoli preserved and regular nuclear membrane. The cytoplasm was well preserved. In the cases classified as atypical, the presence of cell overlapping with nuclear atypia and hyperchromatic nuclei with irregular nuclear membrane was clear. The background of the smears showed up clean, reducing the cell pellet and blood cells, allowing a better position to evaluate the cellular findings.

When the methods of the samples preparation were modified, and the method we are introducing was applied, a decreased number of unsatisfactory samples (65% to 49%) were observed. It is a very important finding, since the number of unsatisfactory samples is reduced and the chances of finding cells modification and identifying lesions in initial stages shall be increased. We presume that the appropriate cell concentration and a better representation of the material in cytological preparation, without background interference, may increase the sensitivity of cytological examination (data not shown). This method increases the chances of identifying atypical cells, since the morphological changes could be better identified and characterized.

**Figure 6:**
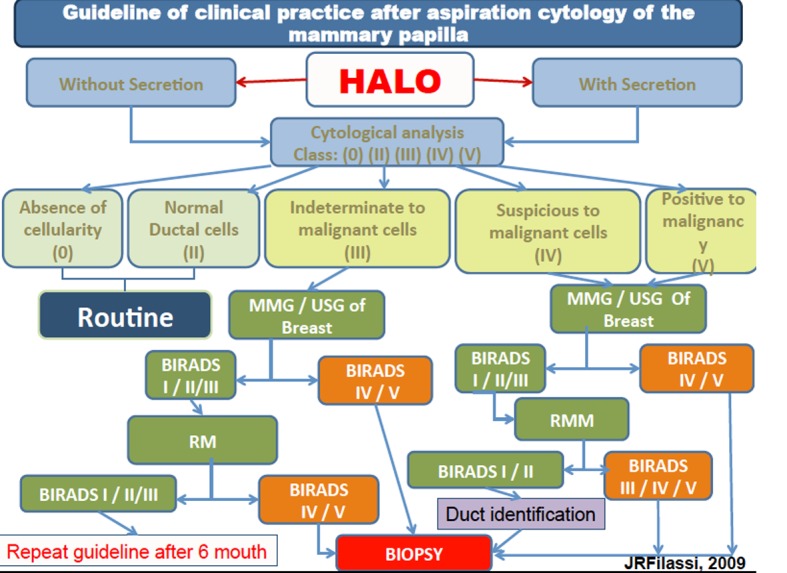
Guideline of clinical practice after aspiration cytology of the mammary papilla.

The presence of atypical cells (class III) in women between 35 and 55 years old was identified in both methods. The identification of atypical cells in women before 40 years old is an important predictor of the presence of premalignant lesion. These results suggest that this method could be an important option to improve the early identification of lesions and guiding the clinician to a possible need to anticipate the search clinic investigation. It is an important finding since the test which is used to prevent breast lesion (mammography) is indicated to women over 40 years old. HMCT shall collect samples in women over 30 years old, increasing the chances to identify lesions in initial stages (Fig. 6).

The alternative methodology we have reported detected 3.0% of atypical cells in contrast to the traditional preparation that showed 1.0%. When the conventional method was modified, the additional centrifugation procedure showed more effectiveness in cell concentration in the sample.

Based on the study of Filassi, 2012, at all, the author suggests a protocol to follow these women as shown below.

### Conclusion

Our study demonstrated an improvement in the quality and quantity of cellular analyses when we associated two methodologies to prepare nipple cytological samples collected, Halo breast test and preserved in liquid medium.
